# Ex Vivo Integration of Human Stem Retinal Ganglion Cells into the Mouse Retina

**DOI:** 10.3390/cells11203241

**Published:** 2022-10-15

**Authors:** Louis-Philippe Croteau, Michael L. Risner, Lauren K. Wareham, Nolan R. McGrady, Xitiz Chamling, Donald J. Zack, David J. Calkins

**Affiliations:** 1 Department of Ophthalmology and Visual Sciences, Vanderbilt Eye Institute, Vanderbilt University Medical Center, Nashville, TN 37232, USA; 2 Department of Ophthalmology, Wilmer Eye Institute, Johns Hopkins University School of Medicine, Baltimore, MD 21287, USA; 3 Department of Genetic Medicine, Johns Hopkins University School of Medicine, Baltimore, MD 21287, USA; 4 The Solomon H. Snyder Department of Neuroscience, Johns Hopkins University School of Medicine, Baltimore, MD 21205, USA; 5 Department of Molecular Biology and Genetics, Johns Hopkins University School of Medicine, Baltimore, MD 21287, USA

**Keywords:** human stem cells, retinal ganglion cell, mouse retina explant, glaucoma, cell replacement therapy

## Abstract

Cell replacement therapies may be key in achieving functional recovery in neurodegenerative optic neuropathies diseases such as glaucoma. One strategy that holds promise in this regard is the use of human embryonic stem cell and induced pluripotent stem-derived retinal ganglion cells (hRGCs). Previous hRGC transplantation studies have shown modest success. This is in part due to the low survival and integration of the transplanted cells in the host retina. The field is further challenged by mixed assays and outcome measurements that probe and determine transplantation success. Thefore, we have devised a transplantation assay involving hRGCs and mouse retina explants that bypasses physical barriers imposed by retinal membranes. We show that hRGC neurites and somas are capable of invading mouse explants with a subset of hRGC neurites being guided by mouse RGC axons. Neonatal mouse retina explants, and to a lesser extent, adult explants, promote hRGC integrity and neurite outgrowth. Using this assay, we tested whether suppmenting cultures with brain derived neurotrophic factor (BDNF) and the adenylate cyclase activator, forskolin, enhances hRGC neurite integration, neurite outgrowth, and integrity. We show that supplementing cultures with a combination BDNF and forskolin strongly favors hRGC integrity, increasing neurite outgrowth and complexity as well as the invasion of mouse explants. The transplantation assay presented here is a practical tool for investigating strategies for testing and optimizing the integration of donor cells into host tissues.

## 1. Introduction

Optic neuropathies diminish the relay of sensory information from the eye to the brain by targeting afferent retinal ganglion cells (RGCs) and their axons, composing the optic nerve. These include both age-related and congenital conditions [1,2]. Chief among age-related conditions is glaucomatous optic neuropathy, or glaucoma, which affects many millions worldwide [3]. Degeneration in glaucoma is progressive and is associated strongly with sensitivity of visual tissues to intraocular pressure [4,5]. Once RGCs and their axons are lost to glaucoma, intrinsic neuronal repair and regeneration is limited [3,6,7,8]. Currently, glaucoma is clinically treated by lowering intraocular pressure, but despite these regimens, visual function often continues to decline as tissue degenerates [9,10]. For these patients, cell replacement strategies hold promise to repair the optic projection and promote restoration of vision [11,12,13].

The last several years have seen great progress in the generation of RGC-like cells from either induced pluripotent or embryonic stem cell origins, though their use in RGC replacement therapies remain in the pre-clinical stage [14,15]. Investigations on transplanting donor RGCs in animal models of optic neuropathy have produced modest success [15]. Major barriers to RGC transplantation include, among others, survival of the grafted cells and integration into host circuitry [15,16]. Transplanted RGCs are further challenged by pro-degenerative environments, immune system responses, and the necessity to project long axons destined for appropriate central targets in the brain. Success in this regard will depend on incorporating many strategies to enhance survival, integration, appropriate axon guidance, and synapse formation of donor RGCs.

At the molecular level, human embryonic stem RGCs (hRGC) appear similar to native RGCs, expressing transcripts for BRN3B, ATOH7, ISL1, and SOX4 [17,18,19]. Moreover, these cells express the RGC-specific protein, RBPMS, and neuron-associated protein, TUJ1 [18,20]. hRGCs produce excitatory inward currents in response to glutamate, and generate action potentials in response to depolarizing stimuli [17,20]. Furthermore, hRGC neurites accumulate excitatory postsynaptic proteins, suggesting the potential to form contacts with other cells [20,21].

Here, we demonstrate a transplantation strategy to test interactions beteween hRGCs and mouse retinal explants, hRGC neurite outgrowth and integrity. We found that hRGC bodies and neurites are capable of cohabitating with retinal explants, with a subset of hRGC neurites displaying affinity for both neonatal and adult mouse RGC axons. Supplementation with brain-derived neurotrophic factor (BDNF) and the adenylate cyclase activator, forskolin, increased hRGC neurite outgrowth through maintaining structural integrity. Interestingly, we provide evidence for the alteration of hRGC neurite maturation by adult mouse retina explants. Our results highlight the importance of exogenous neurotrophic supplementation to promote donor cell neurite outgrowth in adult retinal tissue and the potential for hRGC integration with adult retinal tissues. 

## 2. Materials and Methods

### 2.1. Animals

All experimental procedures were approved by the Vanderbilt University Institutional Animal Care and Use Committee. C57Bl/6 (WT) mice were obtained from Charles River Laboratory (Wilmington, MA, USA).

### 2.2. Coverslip Preparation

One day prior to culture, circular coverslips (Electron Microscopy Sciences, 18 mm #1.5, cat. # 7222201) were coated with Poly-D-lysine (0.5 mg/mL in ultrapure water, Millipore Sigma, St. Louis, MO, USA, cat. # P6407) overnight at room temperature. Coverslips were rinsed with ultrapure water and incubated with laminin (20 μg/mL in PBS, Thermo Fisher, Waltham, MA, USA, cat. # 23017015) for 2 h at 37 °C in a tissue culture incubator. Coverslips were rinsed once with ultrapure water, water was replaced with 200 μL of coating medium (0.2 g of Methyl cellulose (Millipore Sigma, cat. # M0512) in 48.5 mL of Hibernate A (Thermo Fisher, A1247501) and 1 ml of B-27 (Thermo Fisher, cat. #175040440) and 500 μL of L-glutamine (200 mM, Thermo Fisher, cat. # 25030081).

### 2.3. Culture Medium

Culture medium was composed of Neurobasal-A (Thermo Fisher, cat. # 10888022), 2% B-27 supplement (Thermo Fisher, cat. # 17504044), 1% N2 supplement (Thermo Fisher, cat. # 17502048), 0.1% gentamicin (Millipore Sigma, cat. # G1397), 200 mM L-glutamine (Thermo Fisher, cat. # 25030081). With or without the addition of 50 ng/mL recombinant human BDNF (R&D Systems, Minneapolis, MN, USA, cat. # 248-BDB) and 5 μM Forskolin (Stemcell Technologies, Cambridge, MA, USA, cat. # 72114).

### 2.4. Mouse Retina Explant Culture

The mouse retina explant culture protocol was modified from [22]. Briefly, eyes of sacrificed mice were placed in ice-cold PBS. Under a dissection microscope, retinas were extracted and placed in ice-cold Hibernate-A solution. Using #55 Dumont forceps, retinas where cut into roughly 100 pieces, approximately 500 μm in diameter. 5–6 retina explants were transferred to individual coverslips, distributing them roughly homogenously, RGC layer facing down. After letting the explants adhere to the surface for approximately 10 min., the coating medium was removed using a micropipette fitted with a fine PCR tip and explants were allowed to adhere for an additional 5 min. Coverslips containing explants were than transferred to 12 well culture plates (Greiner Bio-One North America, Monroe, NC, USA, cat. # 665180) and 2 mL of room temperature culture media was gently added to wells. Culture plates were than transferred to a cell culture incubator set to 37 °C, 5% CO_2_. The next day, explants that were not adhered were removed, half of the culture media was replaced every other day. Explants were cultured for 48–72 hrs prior to the addition of hRGCs.

### 2.5. Stem Cell Culture

BRN3B-H9 reporter hESCs differentiated by chemical induction toward a RGC fate (hRGCs) were generated by Dr. Donald Zack’s laboratory at Johns Hopkins University [18]. hRGCs were shipped frozen to Vanderbilt University Medical Center and stored in liquid nitrogen upon arrival. Cells were thawed in a 37 °C water bath and plated at the density of 5000 cells/cm^2^.

### 2.6. Immunocytochemistry

Cultures were fixed by adding an equal volume of 37 °C fixative (8% Paraformaldehyde (Electron Microscopy Sciences, Hatfield, PA, USA, cat. # 15714-S), 3% sucrose in PBS) to the volume of culture medium. Cultures were fixed for 20 min at room temperature and washed 3 times with PBS. Primary and secondary antibodies were diluted in a solution of 0.5% Triton X-100, 1% donkey serum in PBS. Cultures were incubated with primary antibodies overnight at 4 °C, washed 3 times with PBS and incubated for 1 hr with secondary antibodies at room temperature, followed by 3 PBS washes. Coverslips were mounted with Fluoromount G (Southern Biotech, Birmingham, AL, USA). The following primary antibodies were used: rabbit anti-RFP (1:1000, Rockland, Pottstown, PA, USA, cat. # 600401379), mouse anti-Tubulin β III (1:1000, Millipore, cat. # MAB5564), goat anti-PSD95 (1:1000, Abcam, Waltham, MA, USA, cat. # ab12093). Secondary antibodies used were Alexa-conjugated donkey anti-rabbit Cy3, anti-mouse 488, anti guinea pig 647, and anti-goat 647, all used at 1:1000.

### 2.7. Imaging

Sixteen-bit tiled images (2.9 × 2.9 mm) were acquired with a Nikon Multi Excitation TIRF equipped with a Andor Xyla sCMOS camera and a Plan Apo VC 20x/0.75 DIC N2 WD = 1.0 mm lens. Images of hRGCs within explants were acquired with a Zeiss LSM800 confocal microscope using a 20x/0.80 Plan-Apochromat, WD = 0.55 mm lens. For conditions including explants, images were captured by setting the explant as the center point (Figure S1A).Image analysis

Images were processed and analyzed with Fiji ImageJ Version 1.53 (NIH). Background subtraction was performed prior to all measurements. To obtain cell counts, DAPI images were binarized followed by a particle analysis set to include particles ranging 15–500 μm^2^ with a circularity of 0.7–1. To obtain hRGC skeleton length measurements, tdTomato images were binarized, pixels were then dilated twice, a Gaussian blur with a sigma value of 10 was applied followed by the application of the skeletonize function. The total length of the skeletonized hRGCs was obtained by running the Summarize Skeleton function, and the average skeleton length was obtained by dividing the total skeleton length by the cell count. Neurite complexity measurements were obtained by quantifying the number of truncated hRGC skeleton branches by running the Summarize Skeleton function, and dividing the number of branches by the cell count. hRGC skeletons were truncated by dilating pixels of binarized DAPI 55 times, generating and combining ROIs of the dilated DAPI signal, inverting the generated combined ROI, and clearing the signal within the inverted ROI in the skeletonized hRGC images. For confocal images of hRGCs within retina explants, maximum Z projections were generated from Z stacks. For the mouse RGC axon/hRGC neurite interaction analysis, hRGCs within 500 µm of explant body edges were excluded from the analysis to avoid potential contact-dependent effects between hRGCs and explants. When a cell body was identified beyond the 500 µm threshold of analysis, only primary outgrowths from the hRGC soma were recorded as events (i.e., intersecting/guided/terminating). For fragmentation analysis, a particle analysis was set to include particles ranging 0.3–20 μm^2^ with a circularity of 0.7–1. A ratio of the area occupied by fragments of tdTomato over the total area of tdTomato was calculated to obtain the fragmentation index. The lower limit of 0.3 μm^2^ was chosen to reduce the inclusion of remaining noise as well as small particle artifacts that occur when imaging regions slightly out of focus. The higher limit of 20 μm^2^ was chosen to exclude small hRGCs without neurite outgrowth. The circularity setting of 0.7–1 was chosen to avoid including small portions of intact neurites that can result from discontinuous tdTomato. For presentation, fragments were dilated 3 times for visualization purposes by using the dilate function in ImageJ. For PSD-95 analysis in neurites, ROIs of cell somas were generated by dilating pixels of binarized DAPI 19 times to approximately cover the cell somas.

### 2.8. Statistical Analysis

We quantified data using Graphpad Version 9 (Graphpad Software LLC., San Diego, CA, USA). We first determined if datasets formed a normal distribution using Shapiro–Wilk tests. If datasets were normally distributed, we performed parametric statistics; otherwise, we performed non-parametric statistics. We defined statistical significance as a *p* value of 0.05 or less.

## 3. Results

### 3.1. Neonatal and Adult Mouse Retina Explants Differentially Enhance hRGC Development In Vitro

**Figure 1:**
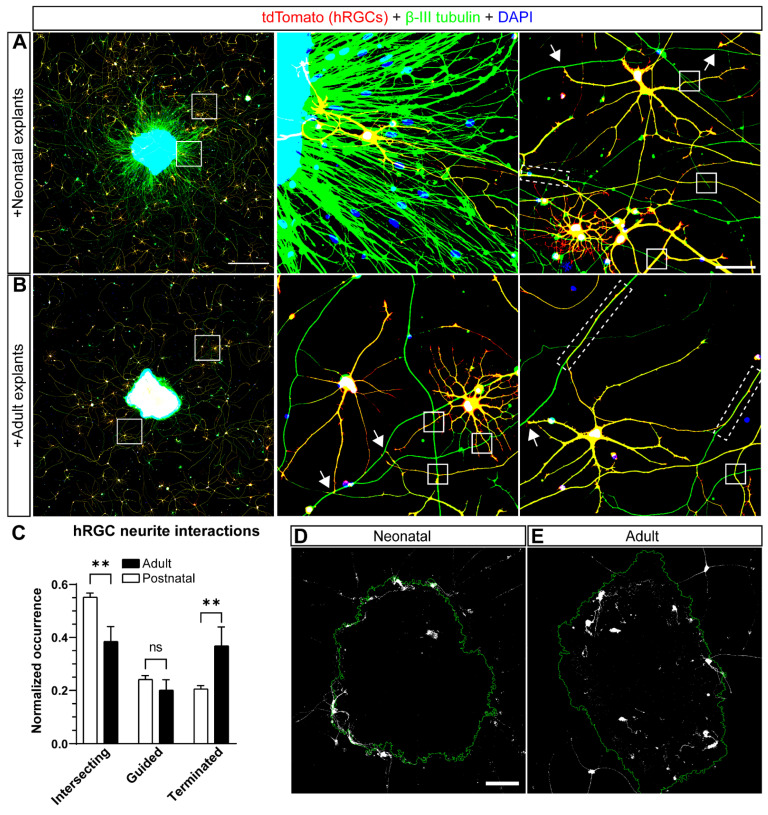
Interactions between hRGCs and mouse retina explants of neonatal and adult age. hRGCs were cocultured for 6 days with either neonatal (P5) or adult (P38) mouse retina explants (**A**,**B**). Cultures were immunostained with an anti-RFP antibody to amplify the tdTomato signal from hRGCs, β-III tubulin to label neurites and DAPI to label nuclei. Middle and right panels show magnified views of the boxes in the left panels. White arrows: examples of hRGC seemingly stalling in proximity to mouse RGC axons (terminated); white squares: examples of hRGCs seemingly disregarding mouse RGC axons (intersecting); white dashed rectangles: hRGC neurites extending upon mouse axons (guided). (**C**) Quantification of the interactions between hRGC primary neurites and mouse RGC axons. The occurrence of hRGC primary neurites intersecting with mouse RGC axons is greater with neonatal explants (*p* = 0.0065). The occurrence of guided events does not differ significantly between neonatal and adult cocultures (*p* = 0.8349). The occurrence of terminated events is greater in the presence of adult explants (*p* = 0.0085) (**D**,**E**) Representative maximum Z projections of confocal images showing the localization of hRGC neurites and cell bodies within mouse retinal explant tissue. Data are shown as mean ± SEM, ** indicates *p* < 0.01, ns = no significance. (**C**) Statistical significance was tested using a 2-way ANOVA, Šidák post hoc test. Neonatal explants: N = 25 2.9 × 2.9 mm images, n = 3–35 hRGCs, adult explants: N = 20 2.9 × 2.9 mm images, n = 1–24 hRGCs. Scale bars: A left = 500 μm, A right = 50 μm, D = 100 μm.

Towards developmenting a strategy to test for hRGC transplantation success, we sought to optimize the chances of hRGC integration. Therefore, we plated hRGCs with retinal explant bodies from both young (P5) and adult (P38) mice cultured in medium supplemented with BDNF and forskolin [23]. We included retinal explants from young mice because postnatal development (P1-14) is marked by extensive plasticity. RGC dendritic arbors expand as postsynaptic densities are refined, the axon initial segment length decreases, indicating changes in excitability, and extraneous RGC bodies are eliminated [24,25,26,27]. Given this period of enhanced plasticity, we reasoned retinal explants from young mice would provide an accommodating environment for interactions between hRGCs and their neurites with explants and RGC axon projections. To test our hypothesis, we compared hRGC neurite interactions when transplanted into mouse neonatal and adult retinal explant bodies. Following 6 days in vitro (DIV), we noted more RGC axons projecting from neonatal explants compared to adult (Figure 1A,B). hRGC neurites interacted with axons projecting from explant bodies in three distinct patterns. Some hRGC neurites seemingly intersected and crossed explant axons without changing their trajectory (termed “intersecting”). In some cases, hRGC neurites ran along explant axons for variable distances before bifurcating (“guided”). Finally, some hRGC neurites abutted axons extending from the explant and stopped (“terminated”). When quantified, the relative occurrence of intersecting hRGC neurites in neonatal retina explants exceeded that of adult explants (+43%, *p* = 0.0065), while the occurrence of terminations was significantly lower (−44%, *p* = 0.0085; Figure 1C). The frequency of hRGC neurite guided events was similar between neonatal and adult explants (*p* = 0.8349, Figure 1C). Finally, many hRGC neurites and somas colocalized with both neonatal and adult mouse retina explants (Figure 1D,E). Some hRGCs located proximal to explants projected neurites within the explant tissue. Conversely, we also observed hRGCs somas within explants that extend neurites outside the explant. The area occupied by hRGCs within mouse retina explant bodies trended higher within adult explants, though not significantly different from neonatal explants (*p* = 0.070).

**Figure 2:**
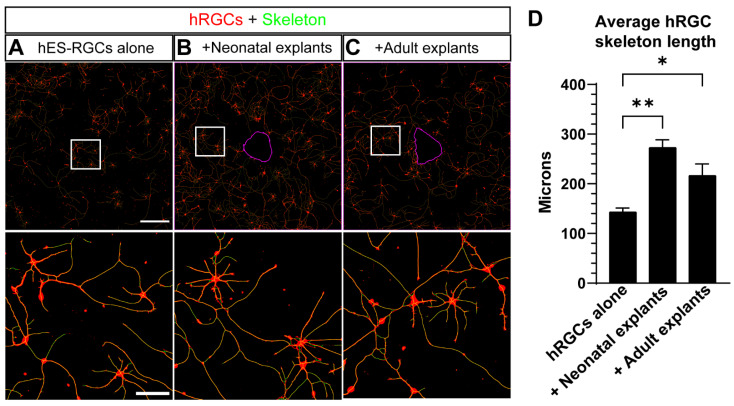
Mouse retina explants increase hRGC neurite outgrowth. (**A**–**C**) Representative images of 6 DIV hRGCs overlayed with their generated skeletons, cultured alone (**A**), with neonatal explants (**B**), and adult explants (**C**). A magenta outline of the explant perimeter is depicted in (**B**,**C**) Bottom panels represent magnified views of the square boxes in top panels. (**D**) The average hRGC skeleton length was quantified by dividing the total length of hRGC skeletons by the number of DAPI+ cells. The presence of both neonatal (*p* = 0.0015) and adult (*p* = 0.0475) increases the average length of hRGC skeletons. hRGCs alone: N = 4 coverslips, n = 4 2.9 × 2.9 mm images, +Neonatal explants: N = 6, n = 3–5, +Adult explants: N = 7, n = 3–5. Scale bars: A top = 500 μm, A bottom = 100 μm. Data are shown as mean ± SEM, * indicate *p* < 0.05, ** indicates *p* < 0.01. Statistical significance was tested using an ordinary one-way ANOVA, Dunnett’s post hoc test.

We next determined the influence of neonatal versus adult retina explants on neurite outgrowth of surrounding hRGCs. We measured the average length of digitally skeletonized hRGC neurites (Figure 2A–C). We found both neonatal (+90%, *p* = 0.0015) and adult explants (+51%, *p* = 0.0475) increased total neurite length of hRGCs compared to hRGC cultured alone (Figure 2D). To ensure our analysis was not biased by the number of cells within each group, we counted the number of DAPI+ cells. We did not detect a significant difference in cell numbers between hRGCs cultured alone or with retinal explants (neonatal explants: *p* = 0.9100, adult explants: *p* = 0.1128, Figure S1B). Our results suggest that neonatal and adult mouse explants are permissive for and promote hRGC neurite outgrowth.

**Figure 3:**
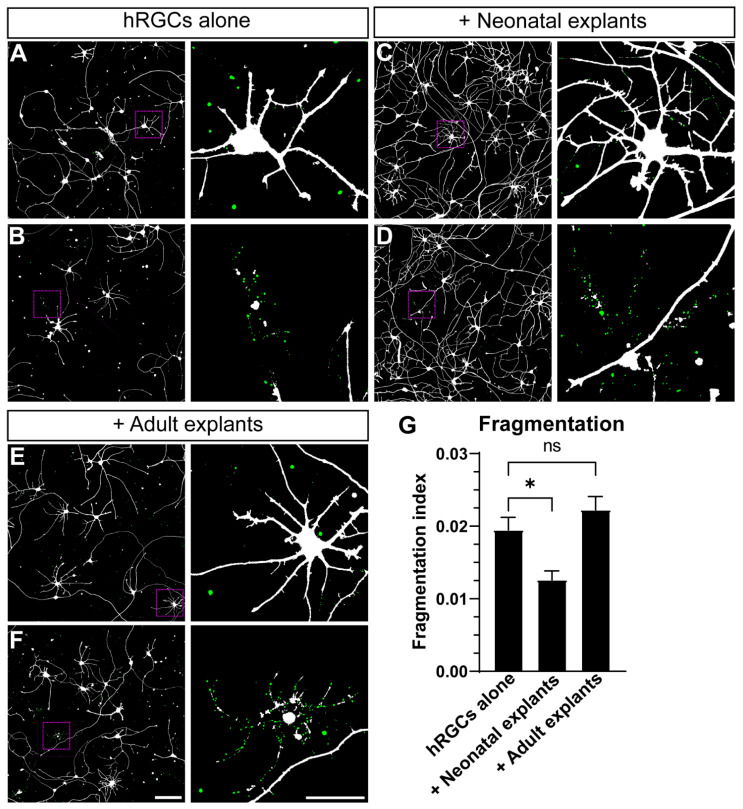
Neonatal explants protect hRGC from fragmentation. (**A**–**F**) Representative images of 6 DIV hRGCs cultured alone (**A**,**B**), with neonatal explants (**C**,**D**), and adult explants (**E**,**F**). For each condition, right panels represent magnified views of the dashed magenta boxes in the left panels. hRGCs fragments have been digitally colorized in green for visualization. (**G**) A quantification of fragmentation reveals lower levels of fragmentation when hRGCs are cocultured with neonatal explants compared to hRGCs cultured alone (*p* = 0.0310). hRGCs alone: N = 4 coverslips, n = 4 2.9 × 2.9 mm images, +Neonatal explants: N = 6, n = 3–5, +Adult explants: N = 7, n = 3.-5. Scale bars: F = 150 μm, F zoom = 50 μm. Data are shown as mean ± SEM, * indicates *p* < 0.05, ns = no significance. Statistical significance was tested using an ordinary one-way ANOVA, Dunnett’s post hoc test.

We assessed the effect of neonatal and adult explants on hRGC degeneration following 6 DIV by measuring the ratio of the area occupied by small neuronal fragments over total area [28,29,30,31]. The hRGC fragmentation index was calculated as the ratio of small tdTomato-positive fragments (size: 0.3–20 μm^2^, circularity:0.7–1) over total tdTomato positive area (Figure 3A–F). We found that fragmentation was lower in the presence of neonatal but not adult explants (−35%, *p* = 0.0438 and +14% *p* = 0.5150, respectively, Figure 3G). These results suggest that neonatal mouse retinal tissue protects hRGC neurites in culture.

### 3.2. Investigating the Influence of Adult Mouse Retina Explants and a Combination of BDNF and Forskolin on hRGCs

**Figure 4:**
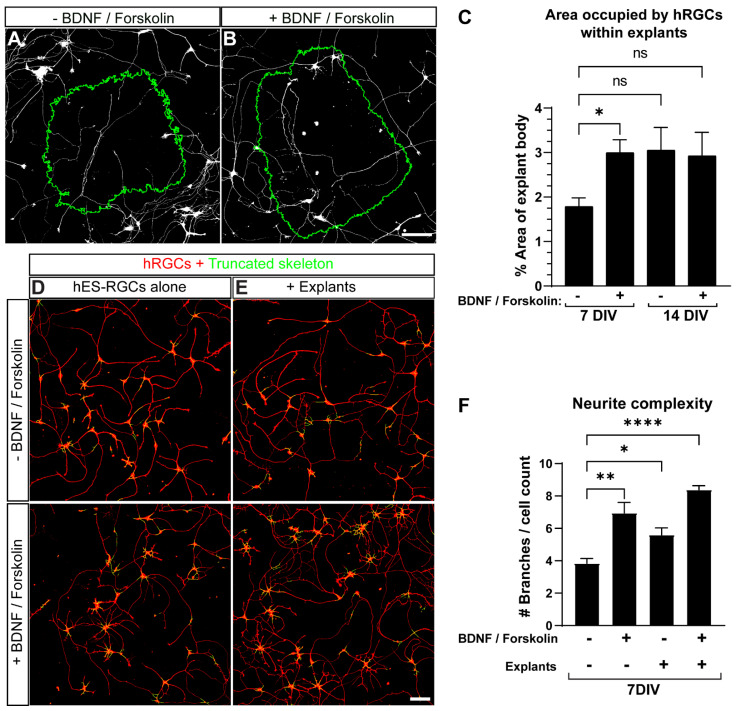
Supplementation with BDNF and forskolin increases the localization of hRGCs within adult mouse retina explants and hRGC neurite complexity. (**A**,**B**) Representative maximum Z projections of confocal images showing the invasion of hRGC neurites and cell bodies within mouse retinal explant tissue after 7DIV without (**A**) and with the addition of BDNF and forskolin (**B**). (**C**) Quantification of the area occupied by hRGCs within mouse retina explants shows that after 7DIV, a combination of BDNF and forskolin increases the area occupied by hRGCs within explant bodies (*p* = 0.0226). At 14 DIV, the area occupied by hRGCs without and with the addition of BDNF and forskolin is not significantly greater than at 7 DIV (*p* = 0.0751 and *p* = 0.0903 respectively). (**D**,**E**) Representative images of 7 DIV hRGCs with their truncated skeletons overlayed, cultured alone (**D**) or cocultured with adult explants (**E**) without (top panels) or with (bottom panels) BDNF and forskolin. Skeletons were digitally truncated to reduce the inclusion of neurite intersects from neighboring cells (**F**) A quantification of neurite complexity measured by counting the number of truncated skeleton branches divided by the number of DAPI+ cells shows that BDNF and forskolin, the presence of explants as well as a combination of BDNF and forskolin with explants, increases neurite complexity (*p* = 0.0017, *p* = 0.0368, *p* < 0.0001 respectively). Scale bars: 100 μm. Data are shown as mean ± SEM, * indicates *p* < 0.05, ** indicates *p* < 0.01, **** indicates *p* < 0.0001. (**C**) Data did not pass the normality test and is shown as Kruskal–Wallis test, (**F**) statistical significance was tested using an ordinary one-way ANOVA, Dunnett’s post hoc test. (**C**) 7DIV hRGC alone N = 13 explants, 7DIV + BDNF, forskolin N = 12, 14DIV hRGC alone N = 9, 14DIV + BDNF, forskolin N = 12. (**F**) hRGCs alone: N = 3 coverslips, n = 3 2.9 × 2.9 mm images, + BDNF, forskolin: N = 3, n = 3, + explants: N = 4, n = 3–4, + explants + BDNF, forskolin: N = 4, n = 3–5.

Although neonatal retinal explant bodies reduced hRGC fragmentation following transplantation compared to adult retinas, the population requiring strong interventions such as cell replacement therapy will typically be adults with age-related optic neuropathies. Thus, we used adult retinal explants and determined the benefit of BDNF + forskolin on hRGC localization and morphology after 7 and 14 DIV (Figure 4A,B). At 7 DIV, BDNF + forskolin produced a 68% increase in area occupied by hRGCs within explants (*p* = 0.0113, Figure 4C). When extending culture duration to 14 DIV, the addition of BDNF + forskolin did not further enhance hRGC localization (*p* ≥ 0.07, Figure 4C). These results suggest BDNF + forskolin enhanced hRGC localization into adult retinal explants. We next evaluated the potential influence of adult mouse retina explants and a combination of BDNF + forskolin on hRGC neurite complexity by quantifying the number of skeletonized hRGC branches. Skeletons of individual cells were truncated to minimize the inclusion of neurites from neighboring cells (Figure 4D,E). Our results show that after 7 DIV, BDNF + forskolin (+81%, *p* = 0.0017), adult explants (+46%, *p* = 0.0368) and a combination of BDNF + forskolin with adult explants (+118%, *p* < 0.0001) increased the average number of skeletonized hRGC branches (Figure 4F).

**Figure 5:**
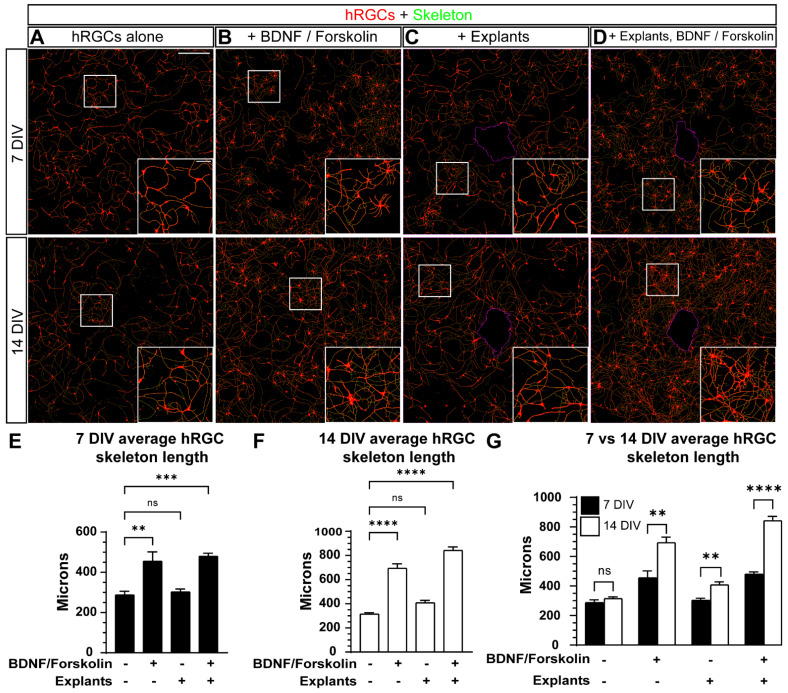
The effect of adult mouse retina explants and a combination of BDNF and forskolin on hRGC neurite outgrowth. (**A**–**D**) Representative images of 7 DIV (top panels) and 14 DIV (bottom panels) hRGCs overlayed with their generated skeletons. hRGCs alone (**A**), with BDNF/forskolin (**B**), cocultured with adult explants (**C**) and cocultured with adult explants and BDNF/forskolin. A magenta outline of the explant perimeter is depicted in (**C**,**D**). Insets depict magnified views of the boxes. (**E**–**G**) A quantification of the average length of skeletonized hRGCs at 7 DIV (**E**), 14 DIV (**F**) and a comparison between 7 and 14 DIV (**G**). At 7 and 14 DIV, both a combination of BDNF and forskolin without and with the presence of adult explants, increases the average hRGC skeleton length (7 DIV: *p* = 0.0025 and *p* = 0.0006, respectively, 14 DIV: *p* < 0.0001). A comparison between the average hRGC skeleton length between 7 and 14 DIV shows that without the addition of BDNF and forskolin or the presence of explants, the average hRGC skeleton length does not significantly differ (*p* = 0.2421). Conversely, supplementation with BDNF and Forskolin, the presence of explants as well as a combination of supplementation with explants, increases the average hRGC skeleton length at 14 DIV compared to 7DIV (*p* = 0.0098, *p* = 0.0067 and *p* < 0.0001 respectively). Scale bars: A top panel= 500 μm, A top panel inset = 100 μm. Data are shown as mean ± SEM, ** indicates *p* < 0.01, *** indicates *p* < 0.001, **** indicates *p* < 0.0001, ns = no significance. Statistical significance was tested using an ordinary one-way ANOVA, Dunnett’s post hoc test (**E,F)** and unpaired t test (**G**). 7DIV hRGCs alone: N = 3 coverslips, n = 3 2.9 × 2.9 mm images, 7DIV + BDNF, forskolin: N = 3, n = 3, 7DIV + explants: N = 4, n = 3–4, 7DIV + explants + BDNF, forskolin: N = 4, n = 3–5, 14DIV hRGCs alone: N = 4, n = 3, 14DIV + BDNF, forskolin: N = 4, n = 3, 14DIV + explants: N = 3, n = 3–4, 14DIV + explants + BDNF, forskolin: N = 4, n = 3–6.

Then, we sought to determine the potential effect of adult mouse retina explants and a combination of BDNF + forskolin on hRGCs neurite extension after 7 and 14 DIV. Quantification of the number of DAPI+ cells indicated that BDNF + forskolin or the presence of explants after 7 and 14 DIV did not alter the cell number compared to hRGCs cultured alone at 7 DIV (Figure S1C). Following microscopy of 7 and 14 DIV cultures, we noticed the addition of BDNF + forskolin appeared to increase hRGC neurite outgrowth relative to hRGC monocultures and retinal explant (Figure 5A,B). As described above, we quantified neurite length by skeletonizing contiguous tdTomato fluorescence, and we found that at 7 and 14 DIV, BDNF + forskolin supplementation significantly increased the average neurite length compared to the control condition (7 DIV: +58%, *p* = 0.0025, 14 DIV: +120%, *p* < 0.0001, Figure 5E,F). Without BDNF + forskolin supplementation, explant did not significantly affect average hRGC neurite length at either time point (7 DIV: *p* = 0.9356, 14 DIV: *p* = 0.1052, Figure 5C,E,F). As expected, hRGC length significantly increased when cultured with explants supplemented with BDNF + forskolin (7 DIV: +67%, *p* = 0.0006, 14 DIV: +167%, *p* <0.0001, Figure 5E,F).

We then determined culture duration affected the average hRGC neurite length within each condition. In hRGC monocultures without supplementation, we did not detect a significant difference in average neurite length between 7 and 14 DIV cultures (*p* = 0.2421, Figure 5G). However, we found the average hRGC neurite length significantly increased over time in monocultures supplemented with BDNF + forskolin (+141%, *p* = 0.0098), in explant cultures without supplementation (+42%, *p* = 0.0067), and the combination of supplements and explants (+75%, *p* < 0.0001, Figure 5G).

**Figure 6:**
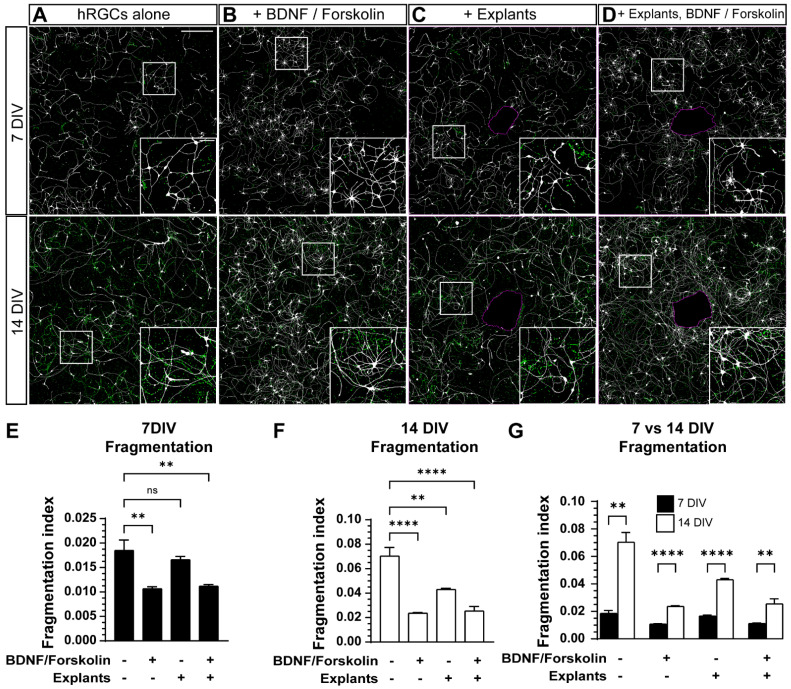
The effect of adult mouse retina explants and a combination of BDNF and forskolin on hRGC integrity. (**A**–**D**) Representative images of 7 DIV (top panels) and 14 DIV (bottom panels) hRGCs and their fragments, digitally dilated and colorized in green for ease of visualization. hRGCs alone (**A**), with BDNF/forskolin (**B**), cocultured with adult explants (**C**), cocultured with adult explants and BDNF/forskolin. A magenta outline of the explant perimeter is depicted in (**C**,**D**). Insets depict magnified views of the boxes. (**E**–**G**) A quantification of fragmentation after 7 DIV (**E**), 14 DIV (**F**) and a comparison between 7 and 14 DIV (**G**). At 7 and 14 DIV, both a combination of BDNF/forskolin without and with the presence of adult explants, decreases hRGC fragmentation (7 DIV: *p* = 0.0010 and *p* = 0.0011, respectively, 14 DIV: *p* < 0.0001). At 14 but not 7 DIV, the presence of explants significantly decreases hRGC fragmentation (*p* = 0.0034 and *p* = 0.4204 respectively). A comparison between fragmentation levels between 7 and 14 DIV shows that fragmentation is greater at 14 DIV in all instances (hRGCs alone: *p* = 0.0017, +BDNF, Forskolin: *p* < 0.0001, +Explants: *p* < 0.0001, +BDNF, Forskolin, Explants: *p* = 0.0088). Scale bars: A top panel = 500 μm, A top panel inset = 100 μm. Data are shown as mean ± SEM, ** indicates *p* < 0.01, **** indicates *p* < 0.0001, ns = no significance. Statistical significance was tested using an ordinary one-way ANOVA, Dunnett’s post hoc test (**E**,**F**) and unpaired t test (**G**). 7DIV hRGCs alone: N = 3 coverslips, n = 3 2.9 × 2.9 mm images, 7DIV + BDNF, forskolin: N = 3, n = 3, 7DIV + explants: N = 4, n = 3–4, 7DIV + explants + BDNF, forskolin: N = 4, n = 3–5, 14DIV hRGCs alone: N = 4, n = 3, 14DIV + BDNF, forskolin: N = 4, n = 3, 14DIV + explants: N = 3, n = 3–4, 14DIV + explants + BDNF, forskolin: N = 4, n = 3–6.

We also explored the effect of adult mouse retina explants and a combination of BDNF + forskolin on hRGCs integrity following 7 and 14 DIV (Figure 6). At 7 DIV, supplementation with BDNF + forskolin in monocultures as well as with retinal explants, decreased the fragmentation index of hRGCs (7 DIV, −42%, *p* = 0.0010, −40%, *p* = 0.0011, respectively, Figure 6E). At 14 DIV, supplementation, the presence of explants, and a combination of supplementation and explants decreased the fragmentation index of hRGCs (−66%, *p* < 0.0001, −39%, *p* = 0.0034, −64%, *p* < 0.0001, respectively, Figure 6G). It is also noteworthy that without supplementation or the presence of explants, fragmentation index was 2.8-fold greater at 14 DIV compared to 7 DIV (*p* = 0.0017, Figure 6G). Taken together, these results suggests that a combination of BDNF + forskolin and to a lesser extent, adult mouse retina explants, increase hRGC neurite outgrowth and are protective against degeneration.

**Figure 7:**
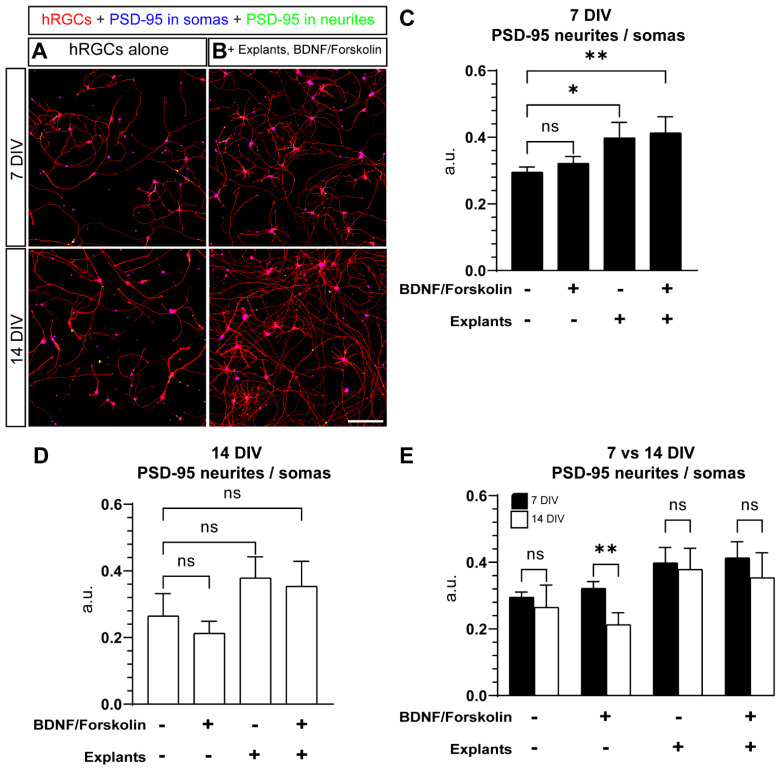
The effect of adult mouse retina explants and a combination of BDNF and forskolin on PSD-95 distribution in hRGCs. (**A**,**B**) Representative images of hRGCs immunostained against PSD-95 with an approximation of somatic and dendritic PSD-95 localization, cultured alone (**A**) or in the presence of adult mouse retina explants and a combination of BDNF/forskolin (**B**) for either 7DIV or 14DIV. (**C**) A quantification of the ratio of the area occupied by PSD-95 in neurites over PSD-95 in somas (PSD-95 neurite/soma) at 7 DIV shows that the presence of adult mouse retina explants without and with the inclusion of BDNF+ forskolin, increases PSD-95 neurites/somas (*p* = 0.0249, *p* = 0.0112 respectively). (**D**) At 14 DIV, supplementation with BDNF, Forskolin or the presence of explants did not significantly alter PSD-95 neurite/soma compared to hRGCs cultured alone. (**E**) A comparison between PSD-95 neurites/somas at 7 and 14 DIV. PSD-95 neurite/soma is lower at 14 DIV when supplemented with BDNF, Forskolin (*p* = 0.0055), Scale bar = 200 μm. Data are shown as mean ± SEM, * indicates *p* < 0.05, ** indicates *p* < 0.01, ns = no significance. Statistical significance was tested using an ordinary one-way ANOVA, Dunnett’s post hoc test. 7DIV hRGCs alone: N = 3 coverslips, n = 3 2.9 × 2.9 mm images, 7DIV + BDNF, forskolin: N = 3, n = 3, 7DIV + explants: N = 4, n = 3–4, 7DIV + explants + BDNF, forskolin: N = 4, n = 3–5, 14DIV hRGCs alone: N = 4, n = 3, 14DIV + BDNF, forskolin: N = 4, n = 3, 14DIV + explants: N = 3, n = 3–4, 14DIV + explants + BDNF, forskolin: N = 4, n = 3–6.

Previously, we found immunofluorescence for the postsynaptic marker, PSD-95, decreases in hRGC somas after 4 weeks and increases in neurites after 3 and 4 weeks compared to 1-week cultures, suggesting maturation of hRGCs over time in culture [20]. Notably, our previous experiments were performed in cultures without BDNF + forskolin supplementation. Here, we tested the influence of BDNF + forskolin, adult retinal explants, and time in vitro on PSD-95 accumulation in hRGCs. We analyzed area occupied by PSD-95 labeling in neurites relative somas (PSD-95 neurite/soma). We did not detect a significant change in PSD-95 neurite/soma in 7 DIV cultures with supplementation alone, (*p* = 0.7276, Figure 7A–C). However, addition of retinal explants with or without BDNF + forskolin significantly enhanced PSD-95 neurite/soma after 7 DIV (+35%, *p* = 0.0144 and +40%, *p* = 0.0064, respectively, Figure 7A–C). Following 14 DIV, retinal explant tended to increase hRGC PSD-95 neurite/soma, but this finding did not meet statistical significance (+Explants: *p* = 0.0853, +Explants, BDNF + forskolin: *p* = 0.1608, Figure 7D). We then sought to isolate the influence of time in vitro on PSD-95 neurite/soma for each condition. In the absence of explants or supplementation or with explants with and without supplementation, we found time in vitro did not affect PSD-95 neurite/soma at this time point (*p* = 0.4844, *p* = 0.6577, *p* = 0.2317, Figure 7E). Interestingly, supplementing monocultures, significantly reduced PSD-95 neurite/soma by 34% (*p* = 0.0055, Figure 7E). Overall, our results suggests that following 7 DIV adult retinal explants may enhance hRGC maturation as indicated by the accumulation of PSD-95 in neurites.

## 4. Discussion

Evidence indicates that hRGCs physically interact with rodent organotypic retinal explants [21,32]. Here, we qualify and quantify how hRGCs interact with neonatal and adult mouse retinal explants. Our results indicate some hRGC neurites seemingly stall or disregard mouse RGC axons, and some extend contiguously with mouse RGC axons for variable distances (Figure 1A,B). Our quantification of the occurrence of hRGC neurite-mouse RGC axon events revealed a greater frequency of intersecting events and a lower frequency of terminating events in neonatal versus adult expants (Figure 1C). The differences we observed may partly be a consequence of the discrepancy in RGC axon density between neonatal and adult explants. However, we attempted to overcome this potential confound by normalizing the number of events by total events. Alternatively, the differences in hRGC interaction with neonatal and adult explants may reflect differences in factors that promote/repel neurite guidance (Figure 1A,B) [33]. In our transplantation assay and analyses, the strength and pattern of electrical activity generated by retinal explant axons may predict the interaction profile of nearby hRGC neurites [34,35]. To avoid the potential confound that cells contacting the explant may behave differently to cells within paracrine signaling distance, we excluded hRGCs within 500 µm of the explant edge from all analyses. Nonetheless, our results indicate a similar frequency of guided events, suggesting that hRGC neurites have comparable affinities for neonatal and adult mouse RGC axons. We also found a similar degree of localization of hRGCs within neonatal and adult mouse explants (Figure 1D,E). Although a tantalizing result, hRGC localization within neonatal explants may be under sampled due to the dense RGC axon outgrowth along the perimeter explant bodies, which might act as a physical barrier (Figure 1A).

Interstingly, when supplemented with BDNF and forskolin, our results suggest that both neonatal and adult retinal explants enhance hRGC neurite outgrowth (Figure 2). Through a quantitative assessment of hRGC fragmentation, we further explored this finding by determining the influence of neonatal and adult retinal explants on hRGC degeneration. We found neonatal retinal explants reduced hRGC fragmentation (Figure 3). This result suggest neonatal retinal tissue reduces degeneration of hRGCs in vitro. However, this measure of fragmentation may indicate neurite remodeling [26]. In support of this idea, we did not detect a significant loss of tdTomato-positive hRGCs between culture conditions (Figure S1B). Follow-up studies will directly compare fragmentation to markers of degeneration and synaptic plasticity in such transplantation strategies.

To determine the influence of BDNF + forskolin on hRGC localization and neurite outgrowth in adult retinal explant over 7–14 DIV, we compared the area occupied by hRGC cultured with explants with and without supplementation. We found BDNF + forskolin significantly enhanced hRGC invasion at 7 DIV compared to explants without supplementation (Figure 4A–C). Similarly, we found adult retinal expant did not significantly impact hRGC skeleton length independent of time in vitro (Figure 5). Regarding the potential engraftment of donor cells into host tissue, the inconsequential affect of adult retinal explants on donor cell neurite outgrowth may be better than a detrimental influence. When supplemented with BDNF + forskolin neurite length is increased with or without explant and regardless of DIV (Figure 5). Based on this finding, cell replacement therapy combined with growth factor supplementation may increase efficacy of donor cell engraftment with host tissue.

Although retinal explant alone did not increase hRGC neurite length (Figure 5), we did observe a beneficial effect of adult retinal explants on hRGCs. hRGCs appeared to intrinsically degenerate from 7–14 DIV as indicated by our fragmentation index (Figure 6G). However, we found retinal explants reduced hRGC degeneration after 14 DIV (Figure 6F). Fragmentation was further reduced with the addition of BDNF + forskolin (Figure 6F). Interestingly, the combination of BDNF + forskolin and explant did not provide an additive effect on fragmentation (Figure 6F). Our results indicate a partially overlapping protective influence of BDNF + forskolin supplementation and adult retinal explants on hRGCs. In regard to BDNF supplementation, evidence suggests chronic exposure to 50 ng/mL BDNF, as used in this study, may promote growth cone collapse [36,37]. Therefore, the concentration of BDNF may need to be modulated in vitro or in vivo to maintain engraftment. In addition, the influence of other factors produced by host tissues on donor cells needs to be thoroughly examined [38].

In the inner plexiform layer of the retina (IPL), RGCs synapse with amacrine and bipolar cells to receive inhibitory and excitatory inputs, respectively [39]. In the rat retina, immunostaining for the postsynaptic marker PSD-95 in the IPL can be seen as early as P0 and reaches an adult staining pattern at P10 [40]. Our quantitative assessment of PSD-95 distribution demonstrates that in the presence of adult explants at 7 DIV, PSD-95 is enriched in neurites, a phenomenon that has also been previously observed in hRGCs over time in culture (Figure 7A–C) [20]. However, our quantificatin of PSD-95 may not represent functional post-synaptic densities. We measured PSD-95 immunolabeling using a 20x objective, which does not provide resolution of punctate PSD-95 labeling. We attempted to overcome this problem by measuring only PSD-95 immunolabeling associated with tdTomato-positive hRGC neurites. Notwithstanding this potential limitation, our finding suggests that adult mouse explants may be secreting factors that influence hRGC neurite maturation/integration. Previous reports lend support to the idea that donor cells can integrate and function in host tissues [41].

Light evoked activity is required for synaptic refinement between RGCs and bipolar cells [42,43]. Furthermore, light stimulation has been shown to enhance RGC regeneration [44]. The transplantation system presented here could be useful in determining the potential effect of light stimulation on enhancing hRGC outgrowth and integration. Future successful RGC replacement therapies will likely be dependent on the axons of grafted replacement cells to use remaining endogenous RGC axons as guidance towards peripheral targets. Through pharmacological and genetic manipulations, the current experimental paradigm may also be useful for investigating strategies for enhancing the affinity of hRGC axons for endogenous axons.

## 5. Conclusions

Retinal ganglion cell replacement in animals indicate modest success due to low survival and integration of donor cells into host retinas. We developed a transplantation assay combining human stem cell-derived RGCs (hRGCs) with mouse retina explants to investigate factors that may optimize the integration of hRGCs into retinal tissue. We found hRGCs invade mouse retina explants with a subset of hRGC neurites guided by mouse RGC axons. BDNF, forskolin, and retina explants enhance hRGC neurite outgrowth and integrity. Our transplantation assay and analytic techniques provide a platform to test mechanisms that may enhance donor cell integration, interactions, and integrity in host tissues.

## Data Availability

Raw data can be requested from the corresponding author.

## Supplementary Materials

The following supporting information can be downloaded at: https://www.mdpi.com/article/10.3390/cells11203241/s1, Figure S1: Supplemental Figure S1.
