# Supplementary material for: Ex Vivo Integration of Human Stem Retinal Ganglion Cells into the Mouse Retina
Source: Cells. 2022 Oct 15;11(20):3241. doi: 10.3390/cells11203241 (PMC9600680; doi:10.3390/cells11203241)
Supplement: Supplementary file 1 [file cells-11-03241-s001.zip › cells-1901791-supplementary.pdf]

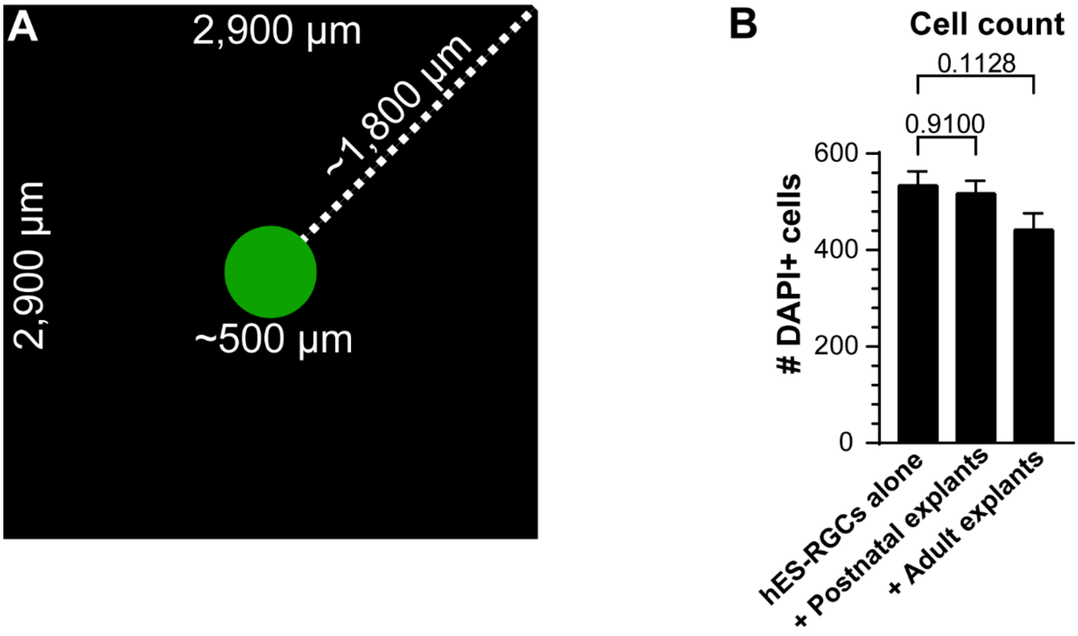

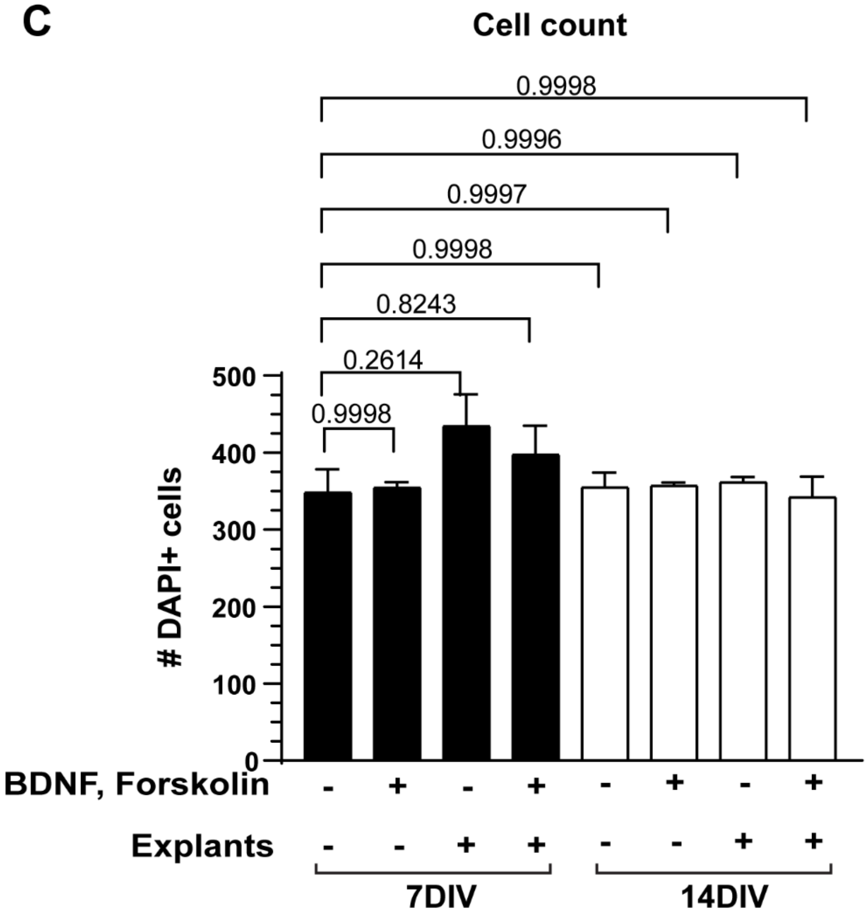

Supplemental Figure S1. (A) Imaging strategy of hRGCs cultured with retinal explants. (**B, C**) Quantification of the number of DAPI+ cells, excluding cells in explants. (**B**) At 6 DIV, there is no difference in cell number with the presence of either neonatal or adult explants. (**C**) At either 7 or 14

DIV, supplementation with BDNF, Forskolin or the presence of adult mouse retina explants does not significantly alter the number of DAPI+ cells compared to hRGCs cultured alone. (**B**) hRGCs alone: N=4 coverslips, n= 4 2.9X2.9mm images, +Neonatal explants: N=6, n=3-5, +Adult explants: N=7, n=3.-5. (**C**) 7 DIV hRGCs alone: N=3 coverslips, n=3 2.9x2.9mm images, 7DIV + BDNF, forskolin: N=3, n=3, 7DIV + explants: N=4, n=3-4, 7DIV + explants + BDNF, forskolin: N=4, n=3-5, 14DIV hRGCs alone: N=4, n=3, 14DIV + BDNF, forskolin: N=4, n=3, 14DIV + explants: N=3, n=3-4, 14DIV + explants + BDNF, forskolin: N=4, n=3-6. Data are shown as mean ± SEM, statistical significance was tested using an ordinary one-way ANOVA, Dunnett's post hoc test.
